# The tyrosine phosphatase SHP2 increases robustness and information transfer within IL-6-induced JAK/STAT signalling

**DOI:** 10.1186/s12964-021-00770-7

**Published:** 2021-09-16

**Authors:** Jessica Fiebelkow, André Guendel, Beate Guendel, Nora Mehwald, Tomasz Jetka, Michal Komorowski, Steffen Waldherr, Fred Schaper, Anna Dittrich

**Affiliations:** 1 Institute of Biology, Department of Systems Biology, Otto-Von-Guericke University Magdeburg, Magdeburg, Germany; 2 Leibniz Institute of Plant Genetics and Crop Plant Research (IPK), Gatersleben, Germany; 3 Karolinska Institutet, Clintec, Huddinge, Sweden; 4 Insilico Medicine, Hong Kong Science and Technology Park, Hong Kong, Hong Kong; 5 Institute of Fundamental Technological Research, Polish Academy of Sciences, Warszawa, Poland; 6 Department of Chemical Engineering, KU Leuven, Leuven, Belgium; 7 Center for Dynamic Systems: Systems Engineering (CDS), Otto‐von‐Guericke University, Magdeburg, Germany; 8 Magdeburg Center for Systems Biology (MACS), Otto‐von‐Guericke University, Magdeburg, Germany

**Keywords:** Signal transduction, SHP2, PTPN11, JAK/STAT, MAPK, Information theory, Channel Capacity, Mutual Information

## Abstract

**Background:**

Cell-to-cell heterogeneity is an inherent feature of multicellular organisms and is central in all physiological and pathophysiological processes including cellular signal transduction. The cytokine IL-6 is an essential mediator of pro- and anti-inflammatory processes. Dysregulated IL-6-induced intracellular JAK/STAT signalling is associated with severe inflammatory and proliferative diseases. Under physiological conditions JAK/STAT signalling is rigorously controlled and timely orchestrated by regulatory mechanisms such as expression of the feedback-inhibitor SOCS3 and activation of the protein-tyrosine phosphatase SHP2 (PTPN11). Interestingly, the function of negative regulators seems not to be restricted to controlling the strength and timely orchestration of IL-6-induced STAT3 activation. Exemplarily, SOCS3 increases robustness of late IL-6-induced STAT3 activation against heterogenous STAT3 expression and reduces the amount of information transferred through JAK/STAT signalling.

**Methods:**

Here we use multiplexed single-cell analyses and information theoretic approaches to clarify whether also SHP2 contributes to robustness of STAT3 activation and whether SHP2 affects the amount of information transferred through IL-6-induced JAK/STAT signalling.

**Results:**

SHP2 increases robustness of both basal, cytokine-independent STAT3 activation and early IL-6-induced STAT3 activation against differential STAT3 expression. However, SHP2 does not affect robustness of late IL-6-induced STAT3 activation. In contrast to SOCS3, SHP2 increases the amount of information transferred through IL-6-induced JAK/STAT signalling, probably by reducing cytokine-independent STAT3 activation and thereby increasing sensitivity of the cells. These effects are independent of SHP2-dependent MAPK activation.

**Conclusion:**

In summary, the results of this study extend our knowledge of the functions of SHP2 in IL-6-induced JAK/STAT signalling. SHP2 is not only a repressor of basal and cytokine-induced STAT3 activity, but also ensures robustness and transmission of information.
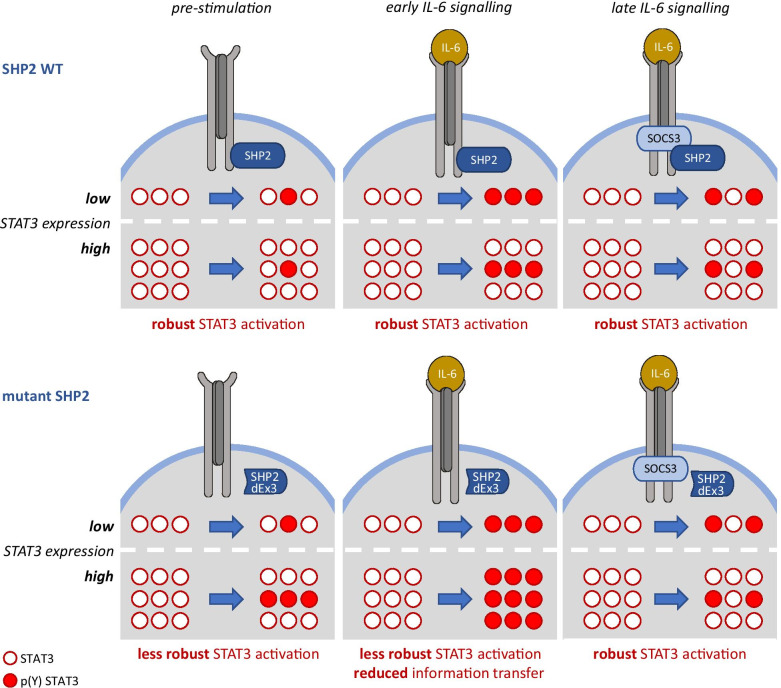

**Plain English summary** Cells within a multicellular organism communicate with each other to exchange information about the environment. Communication between cells is facilitated by soluble molecules that transmit information from one cell to the other. Cytokines such as interleukin-6 are important soluble mediators that are secreted when an organism is faced with infections or inflammation. Secreted cytokines bind to receptors within the membrane of their target cells. This binding induces activation of an intracellular cascade of reactions called signal transduction, which leads to cellular responses. An important example of intracellular signal transduction is JAK/STAT signalling. In healthy organisms signalling is controlled and timed by regulatory mechanisms, whose activation results in a controlled shutdown of signalling pathways. Interestingly, not all cells within an organism are identical. They differ in the amount of proteins involved in signal transduction, such as STAT3. These differences shape cellular communication and responses to intracellular signalling. Here, we show that an important negative regulatory protein called SHP2 (or PTPN11) is not only responsible for shutting down signalling, but also for steering signalling in heterogeneous cell populations. SHP2 increases robustness of STAT3 activation against variable STAT3 amounts in individual cells. Additionally, it increases the amount of information transferred through JAK/STAT signalling by increasing the dynamic range of pathway activation in heterogeneous cell populations. This is an amazing new function of negative regulatory proteins that contributes to communication in heterogeneous multicellular organisms in health and disease.

**Video Abstract**

**Supplementary Information:**

The online version contains supplementary material available at 10.1186/s12964-021-00770-7.

## Background

Interleukin-6 (IL-6) is a central mediator of both pro- and anti-inflammatory responses and has recently obtained further attention due to its prominent role in SARS-CoV2-induced severe cytokine storm [1]. IL6-induced signalling is initiated by binding of IL-6 to the IL-6 receptor α (IL-6Rα), which leads to recruitment of glycoprotein 130 (gp130). The formation of the receptor complex consisting of IL-6, IL-6Rα and gp130, induces activation of gp130-associated Janus kinases (JAK) and subsequent phosphorylation of tyrosine residues within the cytoplasmic part of gp130. These phosphorylated tyrosine motifs serve as binding sites for signal transducers and activators of transcription (STAT). After their recruitment to the receptor STATs are phosphorylated by JAKs, dimerize and translocate into the nucleus [2]. Constitutive, overshooting and prolonged activation of STAT3 is associated with severe inflammatory and proliferative diseases, which has shifted the focus of research on understanding mechanisms of negative regulation of JAK/STAT signalling. The negative feedback inhibitor suppressor of cytokine signalling 3 (SOCS3) inhibits JAK activity and thereby contributes to the transient activation profile of STAT3 [3]. SOCS3 expression is induced in response to STAT3 activation [4] and therefore SOCS3 is only active in late IL-6-induced signalling [5]. In contrast to SOCS3 the cytoplasmic tyrosine phosphatase Src homology region 2 domain-containing phosphatase 2 (SHP2), encoded by the gene *PTPN11*, is expressed constitutively. SHP2 contains two N-terminal SH2 domains followed by a protein-tyrosine-phosphatase (PTP) domain. In the C-terminal part of SHP2 two regulatory tyrosine-residues are located [6]. In the inactive state the N-terminal SH2 domain interacts with the PTP-domain, which blocks PTP activity [6, 7]. Activation of SHP2 is achieved either by binding of the SH2 domains to phosphorylated tyrosine motifs in other proteins, such as phosphorylated Y759 in gp130 [8], or to the two phosphorylated tyrosine motifs within SHP2 itself [9, 10]. In response to IL-6 SHP2 is rapidly recruited to gp130 [8] and phosphorylated by JAKs [11], which hints to a function as early feedback inhibitor. Additionally, SHP2 is a repressor of basal cytokine-independent STAT3 phosphorylation [12, 13]. Beside it´s function as negative regulator of JAK/STAT signalling SHP2 is crucial for IL-6-induced activation of mitogen-activated protein kinase (MAPK) pathway [8, 14]. Binding of SHP2 to the (p)Y759 motif in gp130 and subsequent phosphorylation by JAKs initiates activation of MAPK by recruiting growth factor-receptor-bound protein/son of sevenless (Grb/SOS) complex and/or Grb2-associated binder-1 (Gab1) to the membrane [15–17]. Additionally, SHP2 might increase MAPK activation by binding to Gab1 and/or dephosphorylating binding sides of Ras-GTPase-activating protein (RasGAP) in Gab1 [17, 18].

Protein expression including the expression of signalling proteins is highly heterogeneous even in isogenic cells from the same tissue or organ. This heterogeneity shapes activation and inactivation of signalling pathways. Multiplexed single-cell data and methods from information theory are used to address and quantify consequences of heterogenous protein expression [19]. In information theory, transmission of a signal from a sender to a receiver via a noisy channel is analysed. In terms of signal transduction, the sender can be interpreted as cytokine (or any other upstream signalling molecule), the channel corresponds to the signalling pathway, and an activated transcription factor such as STAT3 (or any other downstream signalling protein) is seen as receiver. Signalling via this channel is shaped by fluctuations in the microenvironment, heterogeneity of protein expression or posttranslational modifications, and stochasticity of protein–protein-interactions. This interpretation allows analysis of the signalling pathways by information theoretic measures such as Mutual Information (MI) and Channel Capacity (CC). MI describes the dependency of two signals, i.e. how much information about one signal can be inferred from knowing the other signal. In advantage over linear regression analysis, MI analysis not only determines linear but also non-linear dependencies that are commonly observed in living systems. We interpret MI as a measure for the robustness of a downstream signal against potential disturbances. A robust signalling event depends on the presence of a stimulus but is independent of biological variation in e.g. copy numbers of signalling proteins. Exemplarily, STAT3 activation is interpreted as robust when it is independent of an individual cell´s STAT3 content.

CC describes the number of distinct response distributions that can be differentiated and thus CC can be interpreted as a metric of pathway’s fitness or potency for a specific condition. Exemplarily, high CC of IL-6-induced STAT3 activation means that different IL-6 concentrations are transduced to differentiable distributions of activated STAT3 and thus allow sensing of different IL-6 doses.

We have recently shown that robustness of IL-6-induced JAK/STAT signalling is achieved by different mechanisms that complement each other at different time scales and cytokine doses. Early JAK/STAT signalling is robust as long as STAT3 phosphorylation is low (e.g. because of low cytokine concentration) and STAT3 expression is high. Robustness of late JAK/STAT signalling is achieved by expression of SOCS3. Channel Capacity of IL-6-induced JAK/STAT signalling in general is restricted by heterogeneity of STAT3 expression and in particular at late time points by expression of SOCS3 [20].

So far, it is not known whether the phosphatase SHP2 affects robustness and Channel Capacity of IL-6-induced signalling. Here, we use multiplexed single-cell flow cytometry data and information theoretic approaches and show that SHP2 increases robustness of basal cytokine-independent STAT3 activation and early IL-6-induced STAT3 activation to differential expression of STAT3 in individual cells. However, in late signalling SHP2 does not affect robustness. In contrast to the feedback-inhibitor SOCS3, SHP2 does not reduce but increases Channel Capacity. This is most probably caused by an increase in the dynamic range of STAT3 phosphorylation through SHP2-dependent inhibition of basal STAT3 phosphorylation.

## Methods

### Cell culture

Generation of SHP2 ΔEx3 mice and immortalised fibroblasts was described earlier by Saxton et al. [21, 22]. In brief, exon 3 in *Ptpn11,* encoding for amino acids 46 – 110, was deleted by homologous recombination. Reconstitution of MEF SHP2 ΔEx3 cells with cDNA for wt SHP2 was described by Oh et al. [22]. MEF wt, MEF SHP2 ΔEx3, and MEF SHP2 ΔEx3 + SHP2 cells were grown in DMEM (Thermo Fisher Scientific, Waltham, MA, USA) supplemented with 10% FCS (Thermo Fisher Scientific), streptomycin and penicillin (each 100 U/ml, Thermo Fisher Scientific) at 37 °C in a water saturated atmosphere containing 5% CO_2_.

### Stimulation of cells

3.5 × 10^5^ cells were cultured on a 3.5 cm dish for 24 h. Prior to stimulation, cells were washed with PBS and subsequently starved for 2 h in 2 ml medium without FCS and antibiotics. Cells were treated with Hy-IL-6 (Conaris, Kiel, Germany) and U0126 (Cell Signaling Technology, Cambridge, UK) as indicated in the figures.

### Western Blotting

For the isolation of cellular proteins, cells were lysed in RIPA lysis buffer (50 mM Tris–HCl, pH 7.4, 150 mM NaCl, 0.5% NP-40, 15% Glycerol, supplemented with 10 µg/ml of each aprotinin, leupeptin and pepstatin as well as 0.8 µM Pefabloc (Roche, Mannheim, Germany), 1 mM NaF, and 1 mM Na_3_VO_4_). The protein concentration of the lysates was determined using Bradford Assay according to manufacturer´s instructions (Carl Roth, Karlsruhe, Germany). Proteins were separated by SDS-PAGE and transferred to a nitrocellulose membrane. Antigens were detected by incubation with specific primary antibodies (1:1,000) followed by incubation with DyLight-coupled secondary antibodies (1:10,000) (Licor, Lincoln, NE, USA). List of primary antibodies: (p)Y SHP2 (#3751), (p)Y STAT3 (#9145), STAT3 (#9139), (p)Y/T ERK1/2 (#4370), ERK1/2 (#4695) (Cell Signaling Technology); SOCS3 (#18391) (Immuno-Biological Laboratories, Fujioka, Japan); HSC70 (#7009) (Stress Marq, Victoria, Canada); SHP2 (#K0810) (Santa Cruz Biotechnology, Dallas, TEX, USA); Tubulin (#T5168) (Sigma-Aldrich Chemie, Munich, Germany). Detection was performed using an Odyssey gel documentation system (Licor). Analysis of Western blots was performed using Image Studio Lite (version 5.2, Licor).

### Flow cytometry

MEF cells were starved for 2 h in DMEM without FCS and antibiotics. After stimulation with Hy-IL-6 and U0126 as indicated, cells were detached from the cell culture dish with 300 µl Trypsin (Thermo Fisher Scientific). For intracellular staining cells were fixed. Therefore, 100 µl of the cell suspension was mixed with 100 µl paraformaldehyde (4%) and incubated at 37 °C for 10 min followed by centrifugation at 300 g, 4 °C for 5 min. Cell pellets were suspended in ice cold 90% methanol and incubated at -20 °C for 10 min. Subsequently, cells were washed twice with ice-cold BSA-EDTA-Buffer (1% BSA, 2 mM EDTA in PBS) and incubated with saturated fluorophore-coupled primary antibodies against STAT3 (#560391) (1:50 in 2% BSA, 2 mM EDTA in PBS) and (p)Y STAT3 (#557814) (1:200 in 2% BSA, 2 mM EDTA in PBS) (BD Biosciences, Franklin Lakes, NJ, USA) overnight. Cells were washed again for three times in 1% BSA-EDTA Buffer before FACS analysis. Analysis was performed using a FACS Canto II (BD Biosciences). Data were analysed using FlowJo (version 10.6.1., BD Biosciences). Specificity of antibodies was validated in STAT3-deficient MEF cells and confirmed absence of unspecific binding (Additional file 1: Figure 1).

### Mutual Information

Mutual Information is used to analyse how much information can be inferred about a random variable by measuring another random variable. It was computed by the formula [23]:

$$MI(S;R)=\iint p(S,R){\mathrm{log}}_{2}\frac{p(S,R)}{p(S)p(R)}dRdS$$

*R* and *S* denote the two random variables to be analysed, in our case the extent of STAT3 phosphorylation and STAT3 expression, respectively. *p(S,R)* is the joint probability density of these two variables and *p(S)* and *p(R)* are the marginal probability densities of the respective variables.

The computation was done with a custom-made Python script using the statistics and integration modules in the "scipy" package (version 0.15.1, Enthought, Austin, TX, USA). All probability densities were approximated by kernel density estimation [24], and the integration was performed with the "quadpack" library. Custom Code for calculation of Mutual Information can be accessed at: https://github.com/swaldherr/il6-heterogeneity.

### Channel Capacity

We employ the most generic model of information transmission, according to which a signal, denoted by S, is transduced by a channel to generate a cellular response, denoted by R [23]. Then, Channel Capacity is defined as the maximal Mutual Information over all possible distributions of the signal p(S)

$$CC(S;R)={\mathrm{max}}_{\mathrm{p}\left(\mathrm{S}\right)}MI\left(S;R\right),$$

where, as in the definition of Mutual Information, it is measured in bits and *S* and *R* are random variables. We measured CC between stimulation level of Hy-IL-6 (S) and the amount of phosphorylated STAT3 (R). Firstly CC can be treated as the maximal (potential) information transfer between S and R. Secondly, the value ${2}^{CC}$ can be interpreted as the number of states of S that can be distinguished with high confidence using knowledge of the values of R [25, 26].

Channel Capacity has been estimated in R (R version 3.6.0) using CRAN package: SLEMI (https://CRAN.R-project.org/package=SLEMI), https://github.com/TJetka/LogRegCapacity which is based on statistical learning methods and Monte Carlo for efficient computation, described and validated elsewhere [27].

### Statistics

Matlab 2019b routines were used for statistical analysis. In order to identify the applicability of parametric testing all tested data sets (MI and CC) were tested for normal behaviour. MI and CC were tested for the impact of genotype, concentration and activation time with 3 to 6 replicates in each data set per subgroup (singular genotype and concentration or activation time). Normal signal behaviour was tested by mean centring each group, effectively eliminating trend variation. Mean centred data was further normalised by division with its standard deviation. Kolmogorov – Smirnov testing using the ks-test function was applied to identify normal distributed data, a requirement for applying parametric tests like Anova. This normalisation allows for multiple groups to be assessed together since group variations are equalized and data trends excluded while the number of individuals is increased to allow more robust test results.

MI data was tested for effects of genotype and concentration of Hy-IL-6 using N-way Anova. The same test was performed for CC in regard to genotype and activation time related effects. Individual groups were then directly compared by 1-way Anova and Effect Size estimation according to unbiased Cohen’s d, constituting the post – hoc testing scheme.

$$d=\frac{{m}_{A}-{m}_{B}}{\sigma }$$

$${d}_{unb}=\frac{{m}_{A}-{m}_{B}}{{s}_{AB}}; {s}_{AB}=\sqrt{\frac{{SS}_{A}+{SS}_{B}}{{df}_{A}+{df}_{B}}}$$

The Effect Size d is a standardized score for the mean difference between two groups A and B using the population standard deviation σ as normalisation factor [28]. To account for different sample sizes and variance in A and B, σ can be substituted by the square root of the sum of the sum of squares (SS_A_ and SS_B_) divided by the sum of degrees of freedom (df) [29].

While the global analysis of the entire population by N-way Anova indicates which factors play a significant role, the further individual assessment of subgroups by 1-way Anova combined with Effect Size estimates underlines trends within factors, like c(Hy-IL-6) or activation time. Significance levels were set to p-values <  = 0.05. Once this comparison criterion was met effect size levels with d > 0.2, d > 0.5 and d > 0.8 indicate small, medium and large effects respectively.

## Results

### SHP2 decreases IL-6-induced STAT3 activation

Cellular heterogeneity e.g. in protein expression and activation is a central feature of multicellular organisms. We have recently shown that the negative feedback inhibitor SOCS3 increases robustness of late IL-6-induced STAT3 activation against differential STAT3 protein expression in individual cells. In contrast, SOCS3 does not affect robustness of early IL-6-induced STAT3 activation or basal cytokine-independent STAT3 activation. Additionally, STAT3-Y705 phosphorylation is robust when cytokine doses are low. In accordance, STAT3-S727 phosphorylation, which results in a reduction of STAT3-Y705 phosphorylation, increases robustness of IL-6-induced STAT3 activation [20]. These observations led us to the overarching hypothesis that negative regulation increases robustness of IL-6-induced STAT3-Y705 phosphorylation against varying STAT3 expression in individual cells. However, the influence of other negative regulatory proteins such as phosphatases on IL-6 signalling in heterogeneous cell populations has not been addressed so far. Consequently, we ask here whether and how the protein-tyrosine-phosphatase SHP2 controls robustness of IL-6-induced STAT3 activation.

We made use of murine embryonal fibroblasts (MEF) expressing a mutant SHP2 protein lacking 65 amino acids within the N-terminal SH2-domain (ΔEx3) [21]. This mutation prevents recruitment of SHP2 to the phosphorylated tyrosine motif 759 within gp130, thereby mimicking a SHP2 knock-out. Expression of SHP2 ΔEx3 enhances IL-6-induced activation of STAT3 and increased activation of STAT3-responsive promoter elements [13].

MEF cells, like most other cells, do not express transmembrane IL-6Rα. Hence, IL-6-induced STAT3 activation in MEF cells depends on trans-signalling enabled by IL-6:soluble IL-6 receptor α (sIL-6Rα) complexes. IL-6 and sIL-6Rα form noncovalent complexes whose equilibrium concentration is not trivially predictable. As a remedy for analyses of dose-dependent effects of IL-6 trans-signalling, Hyper-IL-6 (Hy-IL-6), a fusion protein of IL-6 and sIL-6Rα [30], is used.

**Fig. 1:**
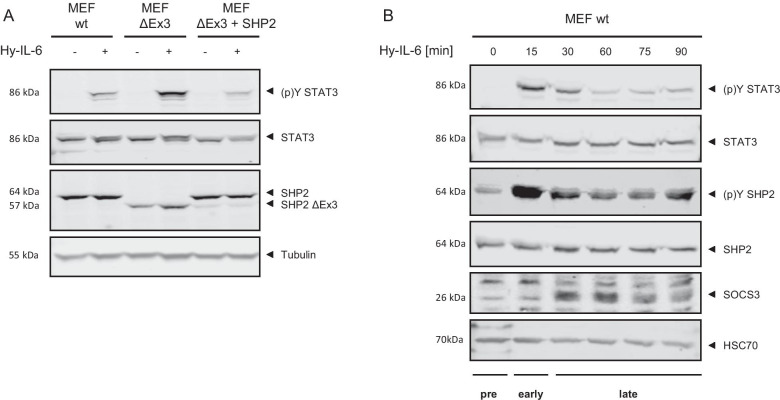
SHP2 reduces IL-6-induced STAT3 phosphorylation. **a** MEF wt, MEF SHP2 ΔEx3, and MEF SHP2 ΔEx3 + SHP2 cells were stimulated with 10 ng/ml Hy-IL-6 for 30 min. **b** MEF wt cells were stimulated with 10 ng/ml Hy-IL-6 for the indicated times. STAT3-Y705, SHP2-Y542 phosphorylation and SHP2, STAT3, SOCS3, Tubulin, and HSC70 expression were evaluated by Western Blotting. Representative results of n = 3 experiments are shown

In accordance with previously published results [13], Hy-IL-6-induced STAT3-Y705 phosphorylation is increased in MEF SHP2 ΔEx3 cells compared to MEF wildtype (wt) cells (Fig. 1a). Mutant cells reconstituted by stable expression of wt SHP (MEF SHP2 ΔEx3 + SHP2) do not show enhanced Hy-IL-6-induced STAT3 phosphorylation. This clearly supports that functional SHP2 is a negative regulator of IL-6-induced STAT3 activation.

Hy-IL-6-induced STAT3-Y705 phosphorylation is transient (Fig. 1b), which underlines the importance of negative inhibitors in shaping signalling and cellular answers in response to IL-6. SOCS3 is not expressed in the first 15 min but after 30 min of Hy-IL-6 stimulation, which coincides with reduced STAT3-Y705 phosphorylation. In contrast to SOCS3, SHP2 is expressed constitutively, while it is phosphorylated transiently in response to Hy-IL-6. Based on these results JAK/STAT signalling can be divided into a pre-stimulation phase, which is independent of IL-6, an early phase with strong STAT3-Y705 phosphorylation and a late phase with low steady state STAT3 activation and expression of SOCS3. Of note, SHP2 is expressed in all three phases.

### SHP2 increases robustness of basal and early IL-6-induced STAT3 activation

Previous analyses revealed that mechanisms enabling robustness of IL-6-induced STAT3 phosphorylation operate in a time- and cytokine-dose-dependent manner [20]. We therefore also analysed the contribution of SHP2 to robustness of STAT3 activation in unstimulated cells and stimulated cells at early and late stages of signalling.

In a first step, we tested whether SHP2 contributes to robustness of basal and early IL-6-induced STAT3 activation. We applied a flow cytometric assay that allows for simultaneous analysis of STAT3 expression and STAT3-Y705 phosphorylation in single cells. MEF wt, MEF SHP2 ΔEx3 cells, and MEF SHP2 ΔEx3 + SHP2 cells were left untreated or were stimulated with increasing amounts of Hy-IL-6 for 15 min. Subsequently, cells were fixed and stained with differentially labelled antibodies against STAT3 and Y705-phosphorylated STAT3.

**Fig. 2:**
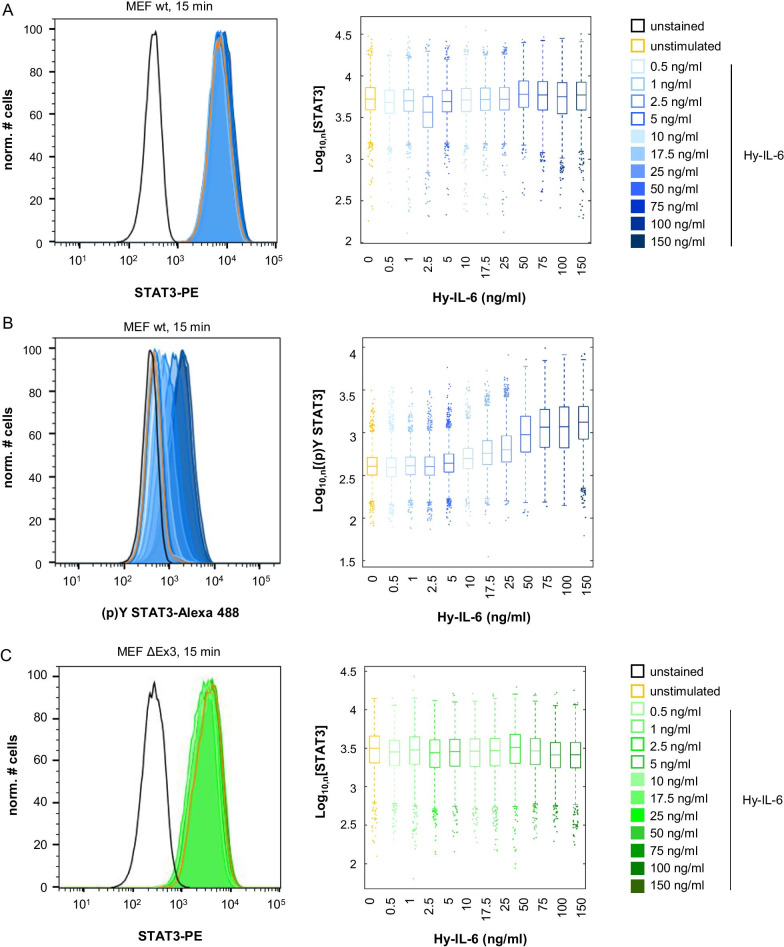

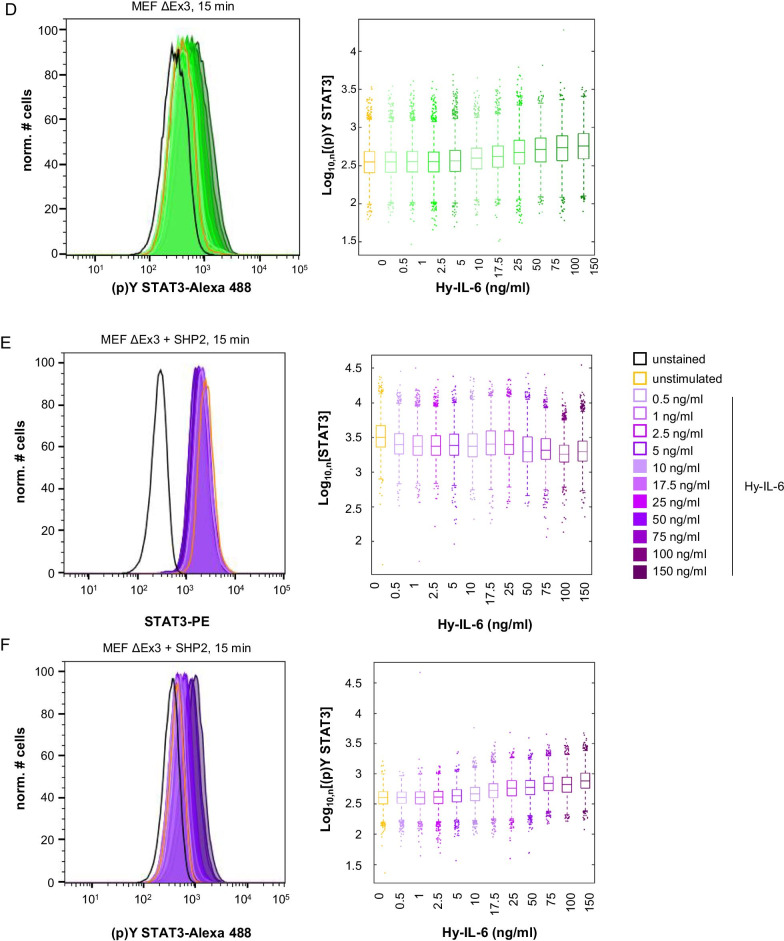
IL-6 signalling results in a dose-dependent heterogeneous STAT3 activation. **a, b** MEF wt cells **c, d** MEF ΔEx3 cells **e, f** MEF ΔEx3 + SHP2 cells were stimulated with increasing amounts of Hy-IL-6 for 15 min. STAT3 expression and phosphorylation were evaluated by intracellular multiplex flow cytometry using specific fluorescent antibodies against STAT3 (A, C, E) and STAT3-Y705 phosphorylation (B, D, F). Representative histograms of n = 3 independent experiments per cell line are shown. Maximal mean fluorescence was normalised to 100%. Log_10_ normalised data points are given as boxplots with median and IQR (box). For better visualisation, only ~ 5% of all data points (n = 1000) per concentration from 3 independent experiments were randomly selected for display

As shown earlier [20] single cell flow cytometry analyses of STAT3 reveal considerable differences in STAT3 expression within the MEF wt cell population, indicating that individual cells differ strongly in respect to STAT3 protein expression (Fig. 2a). The expression and distribution of STAT3 within the cell population are however independent from Hy-IL-6. A 15 min stimulation with Hy-IL-6 results in an increase in STAT3-Y705 phosphorylation (Fig. 2b). Median STAT3 phosphorylation increases dose-dependently up to 75 ng/ml Hy-IL-6. As seen for STAT3 expression STAT3-Y705 phosphorylation varies strongly within the cell population (Fig. 2b). Likewise, in MEF SHP2 ΔEx3 cells and MEF SHP2 ΔEx3 + SHP2 cells STAT3 expression (Fig. 2c, e) and dose-dependent Hy-IL-6-induced STAT3-Y705 phosphorylation (Fig. 2d, f) are heterogeneous.

We next asked to what extent an individual cell´s STAT3 amount affects STAT3-Y705 phosphorylation.

**Fig. 3:**
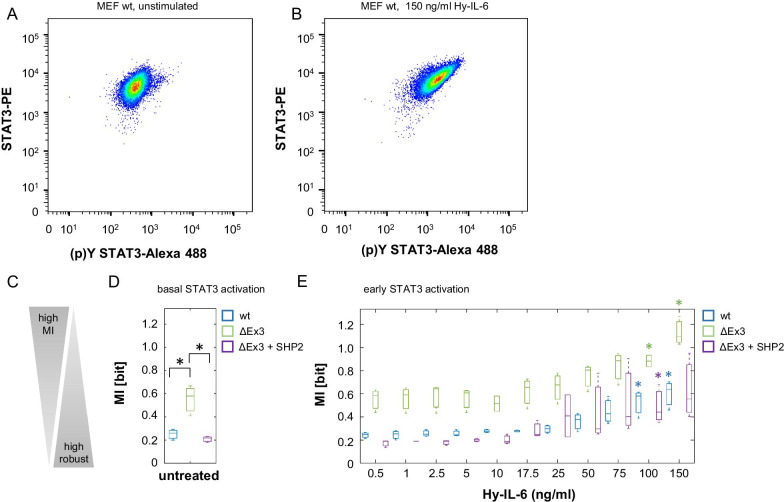
SHP2 increases robustness of basal and early IL-6-induced STAT3 phosphorylation. **a, b** Single cell STAT3 expression and STAT3-Y705 phosphorylation in unstimulated MEF wt cells (A) and MEF wt cells stimulated with 150 ng Hy-IL-6 per ml for 15 min (B). **c** Robustness negatively correlates with Mutual Information. **d** Based on the data presented in Fig. 2a–f Mutual Information between STAT3 expression and cytokine-independent STAT3-Y705 phosphorylation was calculated in unstimulated MEF wt (blue), MEF SHP2 ΔEx3 (green), and MEF SHP2 ΔEx3 + SHP2 cells (violet). Statistics: Stars indicate significant differences between the cell lines. p-value < 0.05 (Anova) and d > 0.2 (complete statistics for Fig. 3 in Additional file 2 Table 1) **e** Based on the data presented in Fig. 2a–f Mutual Information between STAT3 expression and cytokine-dependent STAT3-Y705 phosphorylation was calculated in MEF (blue), MEF SHP2 ΔEx3 (green), and MEF SHP2 ΔEx3 + SHP2 cells (violet) stimulated with the indicated amounts of Hy-IL-6 for 15 min. Data are from n = 3 independent experiments. Statistics: Stars indicate a significant increase of MI in Hy-IL-6-treated cells compared to MI in unstimulated cells (Fig. 3d) within each cell line: blue * (wt), green * (ΔEx3), violet * (Δ Ex3 + SHP2) p-value < 0.05 (Anova) and d > 0.2 (complete statistics for Fig. 3 in Additional file 2: Table 1)

The multiplexed single cell analysis performed, enables us to correlate STAT3 expression and STAT3 activation in single cells. Scatter plots of STAT3 expression and STAT3-Y705 phosphorylation in either unstimulated MEF wt cells (Fig. 3a) or MEF wt cells treated for 15 min with a saturating dose of Hy-IL-6 (Fig. 3b) indicate a positive correlation between the amount of STAT3 and the strength of STAT3 phosphorylation. Of note, the positive correlation is much larger in cells treated with Hy-IL-6 (Pearson correlation = 0.718, p-value < 10^–16^) than in unstimulated cells (Pearson correlation = 0.464, p-value < 10^–16^). To formally challenge the difference between unstimulated cells and cells stimulated with a saturating dose of Hy-IL-6, we employed a two-sample Student’s t-test between Pearson’s correlation coefficients obtained from n = 3 biological replicates. Indeed, Hy-IL-6-induced STAT3 phosphorylation correlates statistically stronger with STAT3 expression than basal cytokine-independent STAT3 phosphorylation (Student’s t-test, β = 0.254, p-value = 0.006).

Calculation of linear regression to quantify the dependency between STAT3 expression and activation can fail to detect non-linear associations and thereby might prevent from discovering critical dynamical features of STAT3 activation. To make sure that all types of correlations and not only linear correlation are captured by our analysis, we use the information theoretic measure MI to quantify the dependency between STAT3 and STAT3-Y705 phosphorylation [20]. MI is known to be a sensitive method to detect different patterns of interdependence [31]. In principle, a lower MI means that two variables are less dependent on each other compared to variables with higher MI. Consequently, we interpret a low MI between STAT3 expression and STAT3-Y705 phosphorylation as high robustness of STAT3 activation to varying STAT3 expression, because the strength of STAT3 phosphorylation in this case is not dependent on the STAT3 amount in an individual cell (Fig. 3c).

In unstimulated MEF wt cells MI between STAT3 expression and STAT3-Y705 phosphorylation is low, indicating that the magnitude of basal STAT3 activation is indeed independent from the magnitude of STAT3 protein expression (Fig. 3d). In unstimulated MEF SHP2 ΔEx3 cells the MI between STAT3 expression and STAT3-Y705 phosphorylation is significantly increased compared to MEF wt cells, indicating that SHP2 increases robustness of basal STAT3 phosphorylation in the pre-stimulation phase. Consequently, reconstitution of mutant cells with wildtype SHP2 (SHP2 ΔEx3 + SHP2) restores robustness of STAT3 activation against varying STAT3 expression. This is in line with the hypothesis that regulatory mechanisms, that reduce STAT3 phosphorylation, increase robustness. In summary, SHP2 increases robustness of basal STAT3 phosphorylation against cell-to-cell heterogeneity in STAT3 expression.

Next, we addressed robustness of early IL-6-induced STAT3 activation against varying STAT3 protein copy number. Hy-IL-6-induced STAT3 phosphorylation in MEF wt cells is robust for low cytokine concentrations. With increasing Hy-IL-6 amounts  resulting in stronger phosphorylation of STAT3 (Fig. 2b), MI is significantly increased compared to MI in unstimulated MEF wt cells (Fig. 3d, e, blue boxes and *) [20]. Also, in MEF SHP2 ΔEx3 cells (green boxes and *) and MEF SHP2 ΔEx3 + SHP2 cells (violet boxes and *) robustness of early STAT3 activation is reduced for high Hy-IL-6 concentrations compared to the corresponding unstimulated cells. This indicates that for low cytokine concentrations STAT3 phosphorylation is robust against heterogeneous STAT3 expression.

Furthermore, mutation of SHP2 (MEF SHP2 ΔEx3) significantly reduces robustness of STAT3 activation independently of Hy-IL-6 dose (Fig. 3e, compare blue and green) (Additional file 2: Table 1). Reconstitution with wt SHP2 (MEF SHP2 ΔEx3 + SHP2 cells) restores robustness (Fig. 3e, compare green and violet) (Additional file 2 Table 1). Thus, SHP2 not only affects robustness of cytokine-independent STAT3 activation but also robustness of early Hy-IL-6-induced STAT3 activation.

In summary, SHP2 contributes to robustness of basal, cytokine independent STAT3 activation and to robustness of early IL-6-induced STAT3 activation. This seems to complement the function of SOCS3, which controls robustness of late IL-6-induced STAT3 activation [20].

### SHP2 does not affect robustness of late IL-6-induced STAT3 activation

**Fig. 4:**
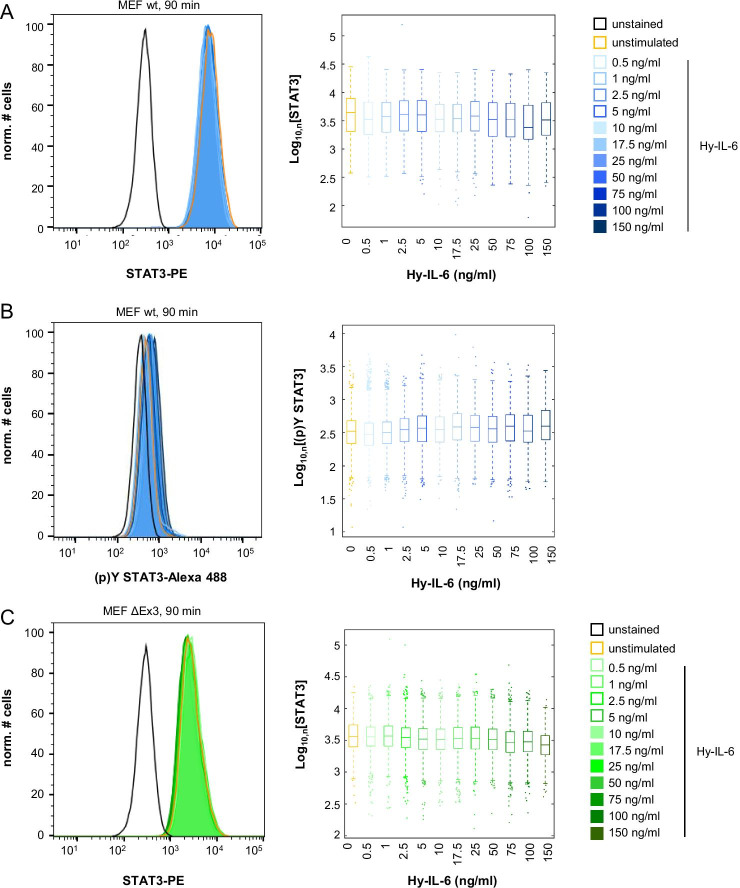

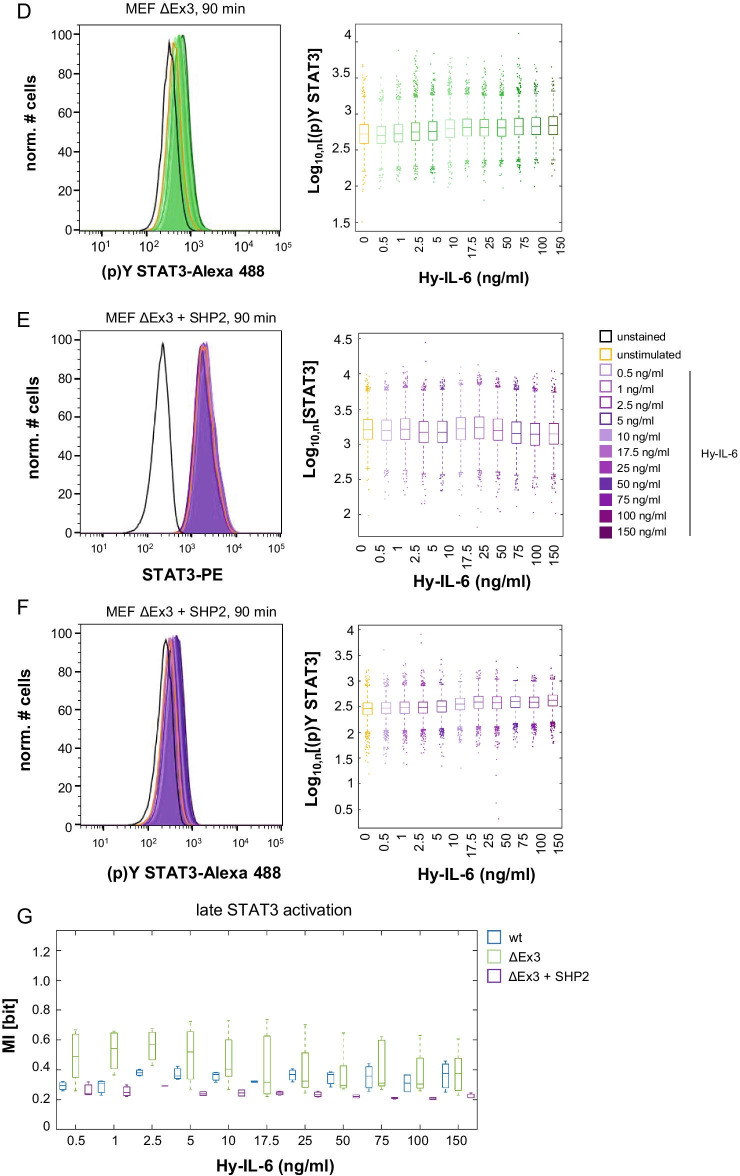
SHP2 does not influence robustness of late IL-6-induced STAT3 phosphorylation. **a, b** MEF wt cells **c, d** MEF ΔEx3 cells **e, f** MEF ΔEx3 + SHP2 cells were stimulated with increasing amounts of Hy-IL-6 for 90 min. STAT3 expression and phosphorylation were evaluated by intracellular multiplex flow cytometry using specific fluorescent antibodies against STAT3 (A, C, E) and STAT3-Y705 phosphorylation (B, D, F). Representative histograms of n = 3 independent experiments are shown. For independent experiments mean fluorescence of cells for each cytokine dose was calculated. Maximal mean fluorescence was normalised to 100%. Log_10_ normalised data points are given as boxplots with median and IQR (box). For better visualisation, only ~ 5% of all data points (n = 1000) per concentration from 3 independent experiments were randomly selected for display. **g** Based on the data presented in Fig. 4 A-F Mutual Information between STAT3 expression and Hy-IL-6-induced STAT3-Y705 phosphorylation in MEF cells (blue), in MEF SHP2 ΔEx3 cells (green), and MEF SHP2 ΔEx3 + SHP2 cells (violet) stimulated with the indicated amount of Hy-IL-6 for 90 min was calculated. Data are from n = 3 - 6 independent experiments. Statistics: no significant differences were observed for p-value < 0.05 (Anova) and d > 0.2 (complete statistics for Fig. 4 in Additional file 3: Table 2)

We next tested whether SHP2 affects robustness of late IL-6-induced STAT3-Y705 phosphorylation. MEF wt (Fig. 4a, b), MEF SHP2 ΔEx3 (Fig. 4c, d), and MEF SHP2 ΔEx3 + SHP2 (Fig. 4e, f) cells were treated with increasing amounts of Hy-IL-6 for 90 min and STAT3 expression and activation were analysed by intracellular multiplex flow cytometry as described above. As seen for short stimulation periods STAT3 expression is heterogeneous in all three cell lines and not affected by Hy-IL-6 (Fig. 4a, c, e). Late Hy-IL-6-induced STAT3-phosphorylation is dose-dependent and heterogenous (Fig. 4b, d, f) but as also shown in Fig. 1B weaker than early STAT3 activation.

In contrast to early STAT3 activation (Fig. 3e) the robustness of late STAT3 phosphorylation is not affected by the concentration of Hy-IL-6 (Fig. 4g), supporting the hypothesis that late STAT3 phosphorylation, which is weaker than early STAT3 phosphorylation (Fig. 1b), is independent from STAT3 expression and thus robust. Interestingly, expression of SHP2 ΔEx3 does not significantly affect robustness of late Hy-IL-6-induced STAT3 activation in contrast to early signalling (Fig. 4g, Additional file 3: Table 2).

In summary, this raises a scenario in which SHP2 and SOCS3 enable robustness of STAT3 activation in a timed manner. SHP2 is expressed constitutively, which enables it to act on basal and early IL-6-induced STAT3 activation. In contrast SOCS3 is not expressed in early signalling (Fig. 1b). When SOCS3 is expressed it reduces STAT3 phosphorylation and increases robustness [20] while SHP2 no longer contributes to robustness.

### Activation of MAPK does not affect robustness of JAK/STAT signalling

**Fig. 5:**
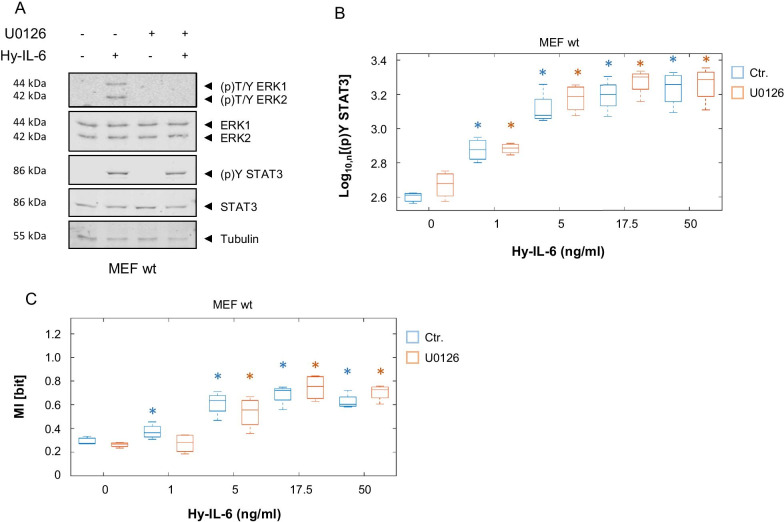
MAPK does not contribute to robustness of IL-6-induced JAK/STAT signalling. **a** MEF wt cells were pre-treated with U0126 (10 µM) for 20 min or left untreated and subsequently stimulated with Hy-IL-6 (20 ng/ml) for 15 min. ERK1/2 and STAT3 phosphorylation and ERK1/2, STAT3, and Tubulin expression were evaluated by Western Blotting. A representative result of n = 3 experiments is shown. **b** MEF wt cells were pre-treated with U0126 (10 µM) for 20 min and subsequently stimulated with the indicated amounts of Hy-IL-6 for 15 min. STAT3 expression and phosphorylation were evaluated by intracellular multiplex flow cytometry using specific fluorescent antibodies against STAT3 and STAT3-Y705 phosphorylation. Mean STAT3 phosphorylation of n = 3 - 4 experiments are shown as boxplots with median and IQR (box). Statistics: no significant differences between control and U0126-treated cells were observed for p-value < 0.05 (Anova) and d > 0.2; stars indicate a significant increase of Hy-IL-6-induced (p)Y STAT3 compared to (p)Y STAT3 in unstimulated cells within each condition: blue * (Ctr.), orange * (U0126), p-value < 0.05 (Anova) and d > 0.2 (complete statistics for Fig. 5b in Additional file 4: Table 3) **c** Based on the multiplexed flow cytometry data Mutual Information between STAT3 expression and Hy-IL-6-induced STAT3-Y705 phosphorylation was calculated. Data are from n = 3 - 4 independent experiments. Statistics: no significant differences between control and U0126-treated cells were observed  for p-value < 0.05 (Anova) and d > 0.2; stars indicate a significant increase of MI in Hy-IL-6-treated cells compared to MI in unstimulated cells within each condition: blue * (Ctr.), orange * (U0126), p-value < 0.05 (Anova) and d > 0.2 (complete statistics for Fig. 5 in Additional file 5: Table 4)

SHP2 reduces robustness of cytokine-independent and early IL-6-induced JAK/STAT signalling against differential STAT3 expression. SHP2 has a dual function in IL-6-induced signalling. While it reduces JAK/STAT signalling it is indispensable for IL-6-induced MAPK pathway activation [32, 33]. It is possible, that SHP2-dependent MAPK activation influences IL-6-induced STAT3 activation in heterogenous cell populations and consequently the robustness of STAT3 activation. Hence, we next asked whether activation of MAPK contributes to robustness of cytokine-independent or early IL-6-induced STAT3 activation. MEF wt cells were treated with the MEK inhibitor U0126 alone or pre-treated with U0126 before stimulation with Hy-IL-6. Activation of MAPK and JAK/STAT signalling was analysed by Western Blotting. IL-6-induced phosphorylation of ERK1/2 was efficiently blocked by U0126, while STAT3 activation was seemingly unaffected (Fig. 5a). Next, U0126 treated and control MEF wt cells were stimulated with increasing amounts of Hy-IL-6. STAT3 expression and phosphorylation were analysed by multiplex intracellular flow cytometry as described before (Fig. 5b). In support of Fig. 5a, the strength of STAT3 phosphorylation induced by both high and low amounts of Hy-IL-6 is independent of the inhibition of the MAPK pathway. Cytokine-independent STAT3 phosphorylation is also not affected by MAPK inhibition (Fig. 5b, Additional file 4: Table 3). Of note, also robustness, as measured by MI between STAT3 expression and phosphorylation, of cytokine-independent and IL-6-induced STAT3 activation against varying STAT3 expression is not affected by inhibition of MAPK (Fig. 5c, compare blue and orange, Additional file 5: Table 4). As shown earlier (Fig. 3e) MI increases significantly with increasing amounts of Hy-IL-6.

In summary, these observations contradict the hypothesis that SHP2-dependent MAPK activation increases robustness of IL-6-induced STAT3 activation in heterogeneous cell populations. SHP2 most probably directly increases robustness of STAT3 activation, independent of MAPK activation.

### SHP2 increases Channel Capacity of IL-6-induced JAK/STAT signalling

**Fig. 6:**
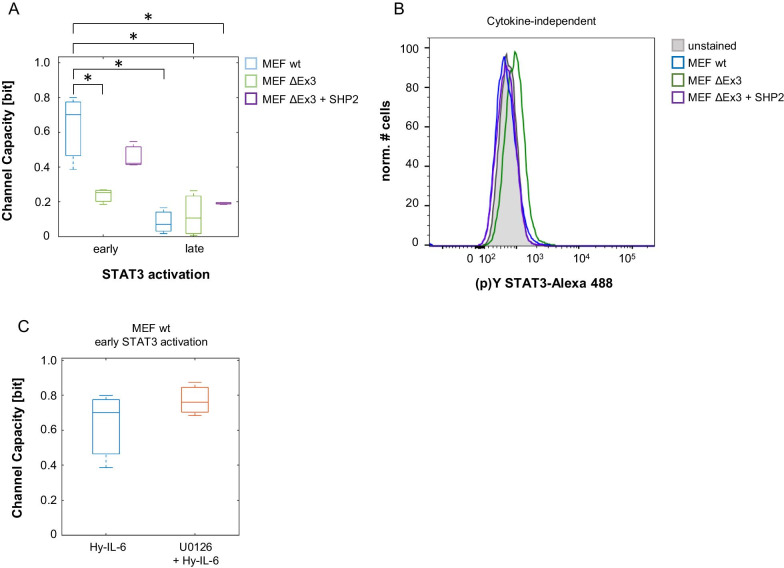
SHP2 increases Channel Capacity of IL-6-induced JAK/STAT signalling. **a** Based on the data presented in Figs. 2 and 4 Channel Capacity of JAK/STAT signalling induced by Hy-IL-6 for 15 min (early) and for 90 min (late) was calculated (MEF wt, blue; MEF SHP2 ΔEx3, green; MEF SHP2 ΔEx3 + SHP2, violet). Data are from n = 3 - 6 independent experiments. Statistics: * p-value < 0.05 (Anova) and d > 0.2 (complete statistics for Fig. 6a in Additional file 6: Table 5) **b** Basal STAT3-Y705 phosphorylation was evaluated in MEF wt (blue), MEF SHP2 ΔEx3 (green), and MEF SHP2 ΔEx3 + SHP2 (violet) cells by intracellular multiplex flow cytometry using specific fluorescent antibodies against STAT3 (p)Y705. Representative histograms of n = 2 independent experiments are shown. **c** MEF wt cells were pre-treated with U0126 (10 µM) for 20 min and subsequently stimulated with increasing amounts of Hy-IL-6 for 15 min. STAT3 expression and phosphorylation were evaluated by intracellular multiplex flow cytometry using specific fluorescent antibodies against STAT3 and STAT3-Y705 phosphorylation (data not shown). Based on these data Channel Capacity of JAK/STAT signalling was calculated (orange) and compared to Channel Capacity of MEF wt cells stimulated with Hy-IL-6 (Fig. 6a, blue). Data are from n = 3 independent experiments. Statistics: no significant differences were observed for p-value < 0.05 (Anova) and d > 0.2 (complete statistics for Fig. 6C in Additional file 7: Table 6)

Channel Capacity is an information theoretic measure for the maximal number of input values - referred here to cytokine concentrations - that can be discriminated by a receiver – referred here to STAT3-Y705 phosphorylation. The negative feedback inhibitor SOCS3 has opposing functions in regulating robustness of STAT3 activation and defining the amount of information transferred through IL-6-induced JAK/STAT signalling. While it increases robustness of STAT3-Y705 phosphorylation it reduces Channel Capacity of late JAK/STAT signalling [20]. We therefore addressed whether the phosphatase SHP2 also affects the amount of information transmitted through IL-6-induced JAK/STAT signalling. To do so we calculated Channel Capacity of early and late IL-6-induced STAT3 activation in MEF wt, MEF SHP2 ΔEx3, and MEF SHP2 ΔEx3 + SHP2 cells based on the data presented in Figs. 2 and 4. Channel Capacity of early Hy-IL-6-induced JAK/STAT signalling in MEF wt cells is approximately 0.7 bit (Fig. 6a, blue). When SHP2 is mutated (green) Channel Capacity is significantly reduced to 0.3 bit. This reduction is partly restored by expression of wt SHP2 (violet), which suggests that SHP2 in contrast to SOCS3 increases information transfer of early IL-6-induced JAK/STAT signalling.

As shown earlier [20] information transfer through the JAK/STAT pathway is strongly reduced at late timepoints, which reflects reduced activation of STAT3 at late time points. Mutation of SHP2 does not affect late Channel Capacity (Fig. 6a).

Interestingly, SHP2 and SOCS3 act opposingly on Channel Capacity. We hypothesize, that SHP2 increases information transfer because it reduces basal STAT3 phosphorylation in the pre-stimulation phase. As a consequence, it would extend the STAT3 phosphorylation range in which cells can operate by increasing their sensitivity to lower stimulation doses. To test this hypothesis, we analysed basal cytokine-independent STAT3 phosphorylation in MEF wt, MEF SHP2 ΔEx3, and MEF SHP2 ΔEx3 + SHP2 cells by intracellular flow cytometry. In line with our hypothesis, basal STAT3-Y705 phosphorylation is increased, when SHP2 is mutated (Fig. 6b).

To test whether SHP2-dependent MAPK affects Channel Capacity of IL-6-induced Jak/STAT signalling, MEF wt cells were pre-treated with the MEK inhibitor U0126 before stimulation with increasing amounts of Hy-IL-6 for 15 min. Expression and phosphorylation of STAT3 were analysed by multiplexed intracellular flow cytometry. Based on these data Channel Capacity of early JAK/STAT signalling was calculated (Fig. 6c, Additional file 7: Table 6) and compared to Channel Capacity of Hy-IL-6 treated MEF wt cells. Inhibition of MAPK does not affect Channel Capacity of Hy-IL-6-induced JAK/STAT signalling, indicating that the amount of information transferred through IL-6-induced JAK/STAT signalling is independent of SHP2-induced MAPK activation.

In summary, our data highlight new functions of the phosphatase SHP2 in ensuring robust STAT3 activity despite heterogeneous STAT3 expression. SHP2 increases robustness and information transfer of early IL-6-induced STAT3-Y705 phosphorylation and ensures independence of basal STAT3 phosphorylation from varying STAT3 expression. However, SHP2 does not affect robustness and information transfer of late IL-6-induced JAK/STAT signalling, indicating a timely orchestration of mechanisms that enable cells to cope with cellular heterogeneity. These effects are most probably independent of SHP2-induced MAPK activation and hence cross-talk of MAPK and JAK/STAT signalling.

## Discussion

The application of information theoretic approaches to intracellular signalling allows to address features of signalling pathways such as robustness, redundancy, and signal amplification that are highly relevant for physiological and pathophysiological signalling [34]. Consequently, these approaches are becoming more and more important for understanding mechanisms of cellular signalling [35, 36]. In contrast to mechanistic systems biology approaches, which rely on detailed knowledge about the architecture of signalling pathways, information theoretic approaches allow the analysis of signalling pathways without detailed knowledge about the underlying molecular mechanisms. Here we use the information theoretic measures Mutual Information and Channel Capacity to define the function of the phosphatase SHP2 as positive regulator of robustness and information transfer of IL-6-induced JAK/STAT signalling.

So far mechanisms of negative regulation of IL-6-induced JAK/STAT signalling were mainly discussed to timely orchestrate signalling and to prevent overshooting signalling which is involved in development of proliferative and chronic inflammatory diseases [2]. To date the most prominent inhibitor of IL-6-induced JAK/STAT signalling is SOCS3. Inhibition of SOCS3 expression [37] or lack of SOCS3 binding sites in gp130 results in sustained STAT3 activation and altered physiological outcomes in response to IL-6 [38]. Additionally, hypermethylation of the SOCS3 promoter and reduced SOCS3 expression is associated with proliferative diseases [39, 40]. The contribution of the phosphatase SHP2 to negative regulation of IL-6-induced JAK/STAT signalling is less well understood. Mutation of SHP2 to prevent recruitment to gp130 or to block phosphatase activity increases basal and IL-6-induced STAT3 activation and STAT3-induced gene expression [13]. Knock down of SHP2 increases basal STAT3 activation [12]. These analyses of negative regulation of JAK/STAT signalling were done in cell populations and did not consider cell-to-cell heterogeneity and its impact on signal transduction. We have recently shown that both STAT3 expression and phosphorylation are highly heterogeneous in isogenic cell populations. This heterogeneity affects robustness and information transfer of IL-6-induced JAK/STAT signalling [20]. Of note, we found that several mechanisms that were so far attributed to negative regulation also affect robustness against cellular heterogeneity. Mechanisms that reduce IL-6-induced STAT3-Y705 phosphorylation such as expression of SOCS3 and STAT3-S727 phosphorylation on the one hand increase robustness of signalling against varying STAT3 expression. On the other hand, these mechanisms reduce the amount of information transferred through IL-6-induced JAK/STAT signalling. This led us to the hypothesis that negative regulatory mechanisms are involved in sustaining robust signalling in the presence of heterogeneous protein expression. Here, we have extended our analyses and to our knowledge for the first time show that the tyrosine phosphatase SHP2 also contributes to robustness of IL-6-induced STAT3 activation in heterogeneous cell populations. Of note, SOCS3 and SHP2 act in a timely orchestrated manner and complement each other. While SHP2 increases robustness of basal and early cytokine-induced STAT3 activation (Fig. 3), SOCS3 increases robustness of late cytokine-induced STAT3 activation. The lack of influence of SHP2 on robustness of late IL-6-induced STAT3 activation (Fig. 4) might be explained by the fact that SHP2 itself is phosphorylated rapidly in response to IL-6 (Fig. 1b) which causes dissociation from gp130 [9–11].

**Fig. 7:**
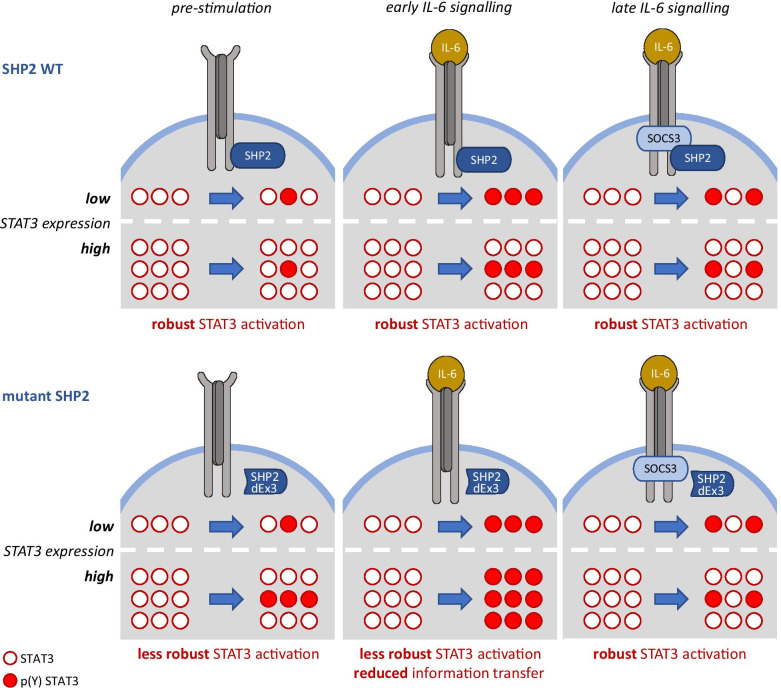
Mutation of SHP2 reduces information transfer and robustness in JAK/STAT signalling. Wt SHP2 contributes to the robustness of STAT3 activation and thus to the independence of the strength of STAT3 activation from varying STAT3 expression, both in unstimulated cells (pre-stimulation) and in cells stimulated with IL-6 for short periods of time (early IL-6-signalling). However, SHP2 does not affect robustness of late IL-6-induced STAT3 activation. Additionally, SHP2 increases the amount of information transferred within early IL-6-induced JAK/STAT signalling. In contrast to SHP2, SOCS3 ensures robustness of late IL-6-induced JAK/STAT signalling.

Although SOCS3 and SHP2 both increase robustness of IL-6-induced STAT3 activation, they affect the amount of information transferred through JAK/STAT signalling in opposite ways (Fig. 7). While SOCS3 reduces Channel Capacity of IL-6-induced JAK/STAT signalling [20], SHP2 increases it (Fig. 6a). This can be explained by the fact, that SHP2 sensitises the cells by reducing basal cytokine-independent STAT3 phosphorylation (Fig. 6b). Cytokine-independent STAT3 phosphorylation adds noise to IL-6-induced STAT3 activation thereby reducing information transfer. Similarly, in epidermal-growth factor (EGF)-induced MAPK signalling, information transfer is increased by negative regulation in the presence of basal activity [41].

We confirmed in this study, that Channel Capacity of IL-6-induced JAK/STAT signalling is below 1 bit (Fig. 6a) [20]. This is often interpreted to mean that an individual cell can merely discriminate between presence and absence of IL-6. This limited information transfer is also described e.g. for growth factor and gonadotropin releasing hormone (GnRH)-induced MAPK signalling [36, 42] and for transforming growth factor β (TGF-β)-dependent SMAD activation [43]. Nonetheless, STAT3 activation as well as activation of MAPK and SMAD are highly dynamic over time. This indicates that a lot of information is potentially encoded in the dynamics of signalling. Indeed, information transfer is higher when dynamics of signalling are considered [35, 44] and more than one output is sensed [23, 27]. Therefore, Channel Capacity analysed at one time-point can be treated as an evidence of much higher cellular capacity as, apparently, the whole dynamical signalling profile is sensed by the cells. Additionally, low information transfer on the single cell level is discussed to be a prerequisite for high information transfer on population level [44, 45].

It is a matter of course, that not only STAT3 expression and activation are heterogenous but also expression and posttranslational modifications of all other proteins and molecules involved in signalling including SHP2 itself. Interestingly, in cell populations the mean amount of SHP2 correlates negatively with the strength of STAT3 activation [12]. Therefore, the amount of SHP2 in an individual cell will probably also affect the strength of STAT3 phosphorylation in this cell. Unravelling this—and infinite other—interplays in single cells, will increase our understanding of the physiological relevance and of both evolutionary advantages and disadvantages of heterogeneity in isogenic cell populations.

In addition to inhibition of JAK/STAT signalling, SHP2 is crucial for activation of IL-6-induced MAPK pathway. Crosstalk between activation of MAPK and JAK/STAT pathways fine-tunes signalling [32]. MAPK are discussed to phosphorylate STAT3 at S727. Serin phosphorylation contributes to reduction of STAT3 Y705 phosphorylation [46], thereby increasing robustness of STAT3 Y705 phosphorylation to varying STAT3 expression [20]. Thus, altered MAPK activation in MEF SHP2 ΔEx3 cells [47] could indirectly contribute to the reduced robustness of STAT3 activation and Channel Capacity in SHP2 mutant cells (Fig. 3). However, pharmacological inhibition of MAPK activation reduces neither robustness of IL-6-induced STAT3 phosphorylation (Fig. 5c) nor Channel Capacity of IL-6-induced JAK/STAT signalling (Fig. 6c). Thus, inhibition of MAPK activity does not reflect the effects observed in MEF SHP2 ΔEx3 cells, indicating that SHP2 does not affect robustness or Channel Capacity indirectly via activation of MAPK. Consistent with these conclusions, IL-6-induced STAT3 tyrosine phosphorylation is not reduced in cell populations after inhibition of MAPK activity (Fig. 5a, b) [16]. Therefore, direct dephosphorylation of members of the JAK/STAT pathway by SHP2 is most likely causative for the described effects. How robustness of MAPK signalling is achieved and whether SHP2 also contributes to robustness and information transfer of MAPK signalling will be addressed in future studies.

SHP2 function is not restricted to IL-6-induced signalling but SHP2 is also a major regulator of signalling induced by other cytokines and growth factors [48]. Loss-of-function as well as gain-of-function mutations and/or overexpression of SHP2 are found in various diseases such as different cancer types [49], Noonan- [50], and LEOPARD-syndrome [51]. Inhibition of SHP2 is seen as a promising strategy for treating several receptor tyrosine kinase-driven cancers [52]. Additionally, interfering with the interaction of SHP2 with programmed death-1 (PD-1) is a potent alternative to immuno-oncologic approaches aiming to block PD-1 with antibodies in several cancers [53]. Based on the results presented here mutations within *PTPN11* and pharmaceutical inhibition of SHP2 probably not only affect strength of intracellular signalling but also induce changes in robustness and information transfer of multiple signalling pathways.

## Conclusion

In conclusion our results extend the knowledge of the functions of SHP2 in the IL-6-induced JAK/STAT signalling pathway from SHP2 as basal repressor and negative regulator to additionally ensuring robustness and information transfer. These results need to be considered for understanding disease-associated SHP2 mutations and for developing pharmaceutical SHP2 inhibitors.

## Supplementary Information

**Additional file 1.Fig. 1** validation of antibodies for flow cytometry.

**Additional file 2.Table 1** (Suppl_Table_1 _Fiebelkow.xlsx), statistics for Fig. 3.

**Additional file 3.Table 2** (Suppl_Table_2 _Fiebelkow.xlsx), statistics for Fig. 4.

**Additional file 4.Table 3** (Suppl_Table_3 _Fiebelkow.xlsx), statistics for Fig. 5b.

**Additional file 5.Table 4** (Suppl_Table_4 _Fiebelkow.xlsx), statistics for Fig. 5c.

**Additional file 6.Table 5** (Suppl_Table_5_Fiebelkow.xlsx), statistics for Fig. 6a.

**Additional file 7.Table 6** (Suppl_Table_6 _Fiebelkow.xlsx), statistics for Fig. 5b.

## Data Availability

The datasets generated and analysed during the current study are available from the corresponding author on reasonable request.

## Abbreviations

CC: Channel Capacity
EGF: Epidermal growth factor
gp130: Glycoprotein 130
GnRH: Gonadotropin releasing hormone
Gab1: Grb2-associated binder-1
Grb: Growth factor-receptor-bound protein
Hy-IL-6: Hyper-IL-6
IL-6: Interleukin-6
IL-6Rα: Interleukin-6 receptor α
JAK: Janus kinase
MAPK: Mitogen-activated protein kinase
MEF: Murine embryonal fibroblasts
MI: Mutual Information
PD-1: Programmed death-1
PTP: Protein-tyrosine-phosphatase
RasGAP: Ras-GTPase-activating protein
SH2: Src homology region 2
SHP2: SH2 domain-containing phosphatase 2
sIL-6Rα: Soluble IL-6 receptor α
STAT: Signal transducer and activator of transcription
SOS: Son of sevenless
SOCS3: Suppressor of cytokine signalling 3
TGF-β: Transforming growth factor β
wt: Wildtype
