# Supplementary material for: The tyrosine phosphatase SHP2 increases robustness and information transfer within IL-6-induced JAK/STAT signalling
Source: Cell Commun Signal. 2021 Sep 16;19:94. doi: 10.1186/s12964-021-00770-7 (PMC8444181; doi:10.1186/s12964-021-00770-7)
Supplement: Supplementary file 2 — Additional file 1.Fig. 1 validation of antibodies for flow cytometry. [file 12964_2021_770_MOESM1_ESM.docx]

**The Tyrosine Phosphatase SHP2 increases Robustness and Information Transfer**

**within IL-6-induced JAK/STAT signalling**

Fiebelkow, Jessica^1^; Guendel, André^2^; Guendel, Beate^1,3^; Mehwald, Nora ^1^, Jetka, Tomasz^4^; Komorowski, Michal^5^; Waldherr, Steffen^6^; Schaper, Fred^1,7,8^; Dittrich, Anna^1,7,8*^

^1^ Institute of Biology, Department of Systems Biology, Otto-von-Guericke University Magdeburg, Magdeburg, Germany

^2^ Leibniz Institute of Plant Genetics and Crop Plant Research (IPK), Gatersleben, Germany

^3^ Karolinska Institutet, Clintec, Huddinge, Sweden

^4^ Insilico Medicine, Hong Kong Science and Technology Park, Hong Kong

^5^ Institute of Fundamental Technological Research, Polish Academy of Sciences, Warszawa, Poland

^6^ Department of Chemical Engineering, KU Leuven, Leuven, Belgium

^7^ Center for Dynamic Systems: Systems Engineering (CDS), Otto‐von‐Guericke University, Magdeburg, Germany

^8^ Magdeburg Center for Systems Biology (MACS), Otto‐von‐Guericke University, Magdeburg, Germany

^*^ corresponding author: anna.dittrich@ovgu.de

**Supplementary Figure**

**Supplementary Figure S1**

**Figure S1: Anti-STAT3 and Anti-(p)Y STAT3 antibodies are specific.**

**A,B)** MEF wt and MEF STAT3^-/-^ cells (generous gift from Valeria Poli) were left untreated (A) or stimulated with Hy-IL-6 (10 ng/ml) for 15 min (B). STAT3 expression and phosphorylation were evaluated by intracellular multiplex flow cytometry using specific fluorescent antibodies against STAT3 (A) and STAT3-Y705 phosphorylation (B). Control cells were left unstained. Representative histograms of n = 3 independent experiments are shown.
